# Supplementary material for: Parameter estimation and identifiability in a neural population model for electro-cortical activity
Source: PLoS Comput Biol. 2019 May 30;15(5):e1006694. doi: 10.1371/journal.pcbi.1006694 (PMC6542506; doi:10.1371/journal.pcbi.1006694)
Supplement: S1 Appendix — The derivation of Eq (8) and detailed expressions for the factors appearing in Eqs (9) and (10). (PDF) [file pcbi.1006694.s001.pdf]

S1 Appendix

Derivation of the transfer function of the system

The full set of the Liley equations involve 14 non-linear integro-differential equations but, with reasonable assumptions about the spatial scales of the variations of the state of the system, can be reduced to an approximate set of 14 partial differential equations [Liley, Cadusch, & Wright, 1999]. For the case in which the long range inputs to the local cortical column can be treated as external given signals, typically of a stochastic nature, these can in turn be approximated by a set of 10 ordinary differential equations describing the local behaviour of the cortical column [Dafilis, Liley, & Cadusch, 2001].

In terms of symbols defined in Table 2 in the main article, these 10 equations are:

$$\tau_e \frac{dh_e}{dt} = h_{er} - h_e + \psi_{ee}(h_e)I_{ee} + \psi_{ie}(h_e)I_{ie} \tag{1a}$$

$$\tau_i \frac{dh_i}{dt} = h_{ir} - h_i + \psi_{ei}(h_i)I_{ei} + \psi_{ii}(h_i)I_{ii} \tag{1b}$$

$$\frac{dI_{ee}}{dt} = V_{ee} \tag{1c}$$

$$\frac{dV_{ee}}{dt} = -2\gamma_e V_{ee} - \gamma_e^2 I_{ee} + (\Gamma_e \gamma_e e N_{ee}^{\beta}) S_e(h_e) + (\Gamma_e \gamma_e e) p_{ee} \tag{1d}$$

$$\frac{dI_{ei}}{dt} = V_{ei} \tag{1e}$$

$$\frac{dV_{ei}}{dt} = -2\gamma_e V_{ei} - \gamma_e^2 I_{ei} + (\Gamma_e \gamma_e e N_{ei}^{\beta}) S_e(h_e) + (\Gamma_e \gamma_e e) p_{ei} \tag{1f}$$

$$\frac{dI_{ie}}{dt} = V_{ie} \tag{1g}$$

$$\frac{dV_{ie}}{dt} = -2\gamma_i V_{ie} - \gamma_i^2 I_{ie} + (\Gamma_i \gamma_i e N_{ie}^{\beta}) S_i(h_i) + (\Gamma_i \gamma_i e) p_{ie} \tag{1h}$$

$$\frac{dI_{ii}}{dt} = V_{ii} \tag{1i}$$

$$\frac{dV_{ii}}{dt} = -2\gamma_i V_{ii} - \gamma_i^2 I_{ii} + (\Gamma_i \gamma_i e N_{ii}^{\beta}) S_i(h_i) + (\Gamma_i \gamma_i e) p_{ii} \tag{1j}$$

To linearize these we restrict attention to fixed points that correspond to stable point attractors. The fixed point equations can be reduced to:

$$V_{ee} = V_{ei} = V_{ie} = V_{ii} = 0 \tag{2a}$$

$$\bar{I}_{ee} = \frac{\Gamma_e e}{\gamma_e}[N_{ee}^{\beta}]S_e(\bar{h}_e) + \left(\frac{\Gamma_e e}{\gamma_e}\right)\bar{p}_{ee} \tag{2b}$$

$$\bar{I}_{ei} = \frac{\Gamma_e e}{\gamma_e}[N_{ei}^{\beta}]S_e(\bar{h}_e) + \left(\frac{\Gamma_e e}{\gamma_e}\right)\bar{p}_{ei} \tag{2c}$$

$$\bar{I}_{ie} = \left(\frac{\Gamma_i e N_{ie}^{\beta}}{\gamma_i}\right)S_i(\bar{h}_i) + \left(\frac{\Gamma_i e}{\gamma_i}\right)\bar{p}_{ie} \tag{2d}$$

$$\bar{I}_{ii} = \left(\frac{\Gamma_i e N_{ii}^{\beta}}{\gamma_i}\right)S_i(\bar{h}_i) + \left(\frac{\Gamma_i e}{\gamma_i}\right)\bar{p}_{ii} \tag{2e}$$

$$0 = h_{er} - \bar{h}_e + \psi_{ee}(\bar{h}_e)\bar{I}_{ee} + \psi_{ie}(\bar{h}_e)\bar{I}_{ie} \tag{2f}$$

$$0 = h_{ir} - \bar{h}_i + \psi_{ei}(\bar{h}_i)\bar{I}_{ei} + \psi_{ii}(\bar{h}_i)\bar{I}_{ii} \tag{2g}$$

where the overbar on a state variable denotes its fixed point value and on an input variable (p_{ee}, p_{ei} etc.) denotes its DC value, treated as a tonic system parameter.

The linearized equations are obtained by expanding the state variables around their fixed point values; the non-zero terms of the corresponding Jacobian, evaluated at the

fixed point, are of the form:

$$J_{11} = \frac{1}{\tau_e}\left(-1 - \frac{\bar{I}_{ee}}{|(h_e^{eq} - h_e^{rest}|} - \frac{\bar{I}_{ie}}{|h_i^{eq} - h_e^{rest}|}\right) \tag{3a}$$

$$J_{13} = \frac{\psi_{ee}(\bar{h}_e)}{\tau_e} \tag{3b}$$

$$J_{17} = \frac{\psi_{ie}(\bar{h}_e)}{\tau_e} \tag{3c}$$

$$J_{22} = \frac{1}{\tau_i}\left(-1 - \frac{\bar{I}_{ei}}{|h_e^{eq} - h_i^{rest}|} - \frac{\bar{I}_{ii}}{|h_i^{eq} - h_i^{rest}|}\right) \tag{3d}$$

$$J_{25} = \frac{\psi_{ei}(\bar{h}_i)}{\tau_i} \tag{3e}$$

$$J_{29} = \frac{\psi_{ii}(\bar{h}_i)}{\tau_i} \tag{3f}$$

$$J_{41} = \Gamma_e \gamma_e e N_{ee}^{\beta} S_e^{'}(\bar{h}_e) \tag{3g}$$

$$J_{43} = -\gamma_e^2 \tag{3h}$$

$$J_{44} = -2\gamma_e \tag{3i}$$

$$J_{61} = \Gamma_e \gamma_e e N_{ei}^{\beta} S_e^{'}(\bar{h}_e) \tag{3j}$$

$$J_{65} = -\gamma_e^2 \tag{3k}$$

$$J_{66} = -2\gamma_e \tag{3l}$$

$$J_{82} = \Gamma_i \gamma_i e N_{ie}^{\beta} S_i^{'}(\bar{h}_i) \tag{3m}$$

$$J_{87} = -\gamma_i^2 \tag{3n}$$

$$J_{10,2} = \Gamma_i \gamma_i e N_{ii}^{\beta} S_i^{'}(\bar{h}_i) \tag{3o}$$

$$J_{10,9} = -\gamma_i^2 \tag{3p}$$

$$J_{10,10} = -2\gamma_i \tag{3q}$$

$$J_{34} = J_{56} = J_{78} = J_{9,10} = 1 \tag{3r}$$

The symbol $S_e^{'}(\bar{h}_e)$ denotes the derivative of $S_e(\bar{h}_e)$ w.r.t. h_e evaluated at the fixed point value $\bar{h}_e$, and similarly for $S_i^{'}(\bar{h}_i)$. Since $J_{43} = J_{65}$ and $J_{87} = J_{10,9}$ and $J_{43} = -\left(\frac{J_{44}}{2}\right)^2$ and $J_{87} = -\left(\frac{J_{88}}{2}\right)^2$ there are only 12 independent elements of the Jacobian viz: the (1,1), (1,3), (1,7), (2,2), (2,5), (2,9), (4,1), (4,4), (6,1), (8,2), (8,8) and (10,2) elements. The resulting structure of the Jacobian is:

$$\begin{bmatrix} J_{11} & 0 & J_{13} & 0 & 0 & 0 & J_{17} & 0 & 0 & 0 \\ 0 & J_{22} & 0 & 0 & J_{25} & 0 & 0 & 0 & J_{29} & 0 \\ 0 & 0 & 0 & 1 & 0 & 0 & 0 & 0 & 0 & 0 \\ J_{41} & 0 & J_{43} & J_{44} & 0 & 0 & 0 & 0 & 0 & 0 \\ 0 & 0 & 0 & 0 & 1 & 0 & 0 & 0 & 0 & 0 \\ J_{61} & 0 & 0 & 0 & J_{65} & J_{66} & 0 & 0 & 0 & 0 \\ 0 & 0 & 0 & 0 & 0 & 0 & 0 & 1 & 0 & 0 \\ 0 & J_{82} & 0 & 0 & 0 & 0 & J_{87} & J_{88} & 0 & 0 \\ 0 & 0 & 0 & 0 & 0 & 0 & 0 & 0 & 0 & 1 \\ 0 & J_{10,2} & 0 & 0 & 0 & 0 & 0 & 0 & J_{10,9} & J_{10,10} \end{bmatrix} \tag{4}$$

The system transfer function can be obtained from the Jacobian in this form, but it is more convenient to rewrite the 10 first-order equations as a set of 2 first order

equations and 4 second order equations. It is also convenient to rewrite them in terms of lumped parameters. The resulting equations are:

$$\tau_e \frac{dh_e}{dt} = h_{er} - h_e + \psi_{ee}(h_e) I_{ee} + \psi_{ie}(h_e) I_{ie} \tag{5a}$$

$$\tau_i \frac{dh_i}{dt} = h_{ir} - h_i + \psi_{ei}(h_i) I_{ei} + \psi_{ii}(h_i) I_{ii} \tag{5b}$$

$$\left(\frac{d}{dt} + \gamma_e\right)^2 I_{ee} = A_{ee} S_e(h_e) + B_{ee} + u_{ee}(t) \tag{5c}$$

$$\left(\frac{d}{dt} + \gamma_e\right)^2 I_{ei} = A_{ei} S_e(h_e) + B_{ei} + u_{ei}(t) \tag{5d}$$

$$\left(\frac{d}{dt} + \gamma_i\right)^2 I_{ie} = A_{ie} S_i(h_i) + B_{ie} + u_{ie}(t) \tag{5e}$$

$$\left(\frac{d}{dt} + \gamma_i\right)^2 I_{ii} = A_{ii} S_i(h_i) + B_{ii} + u_{ii}(t) \tag{5f}$$

The lumped parameters are

$$A_{ee} = (\Gamma_e \gamma_e e N_{ee}^{\beta}) \tag{6a}$$

$$B_{ee} = (\Gamma_e \gamma_e e) \bar{p}_{ee} \tag{6b}$$

$$A_{ei} = (\Gamma_e \gamma_e e N_{ei}^{\beta}) \tag{6c}$$

$$B_{ei} = (\Gamma_e \gamma_e e) \bar{p}_{ei} \tag{6d}$$

$$A_{ie} = (\Gamma_i \gamma_i e N_{ie}^{\beta}) \tag{6e}$$

$$B_{ie} = (\Gamma_i \gamma_i e) \bar{p}_{ie} \tag{6f}$$

$$A_{ii} = (\Gamma_i \gamma_i e N_{ii}^{\beta}) \tag{6g}$$

$$B_{ii} = (\Gamma_i \gamma_i e) \bar{p}_{ii} \tag{6h}$$

The $u_{ee}(t)$ etc. are the time varying parts of the inputs to the system. In practice we usually assume (on physiological grounds) that $B_{ii} = B_{ie} = 0$ and, since, typically, the dominant input to a cortical column is excitatory to excitatory, we also assume that $u_{ei}(t) = u_{ie}(t) = u_{ii}(t) = 0$. Also on physiological grounds, the output of the system (the local EEG signal) is assumed to be proportional to $h_e(t)$.

The 10D system spectrum.

The 10D equations with the coefficient assignments above lead to a simplification of the spectrum. Using the mixed first and second order form, the Laplace transformed linearized equations become:

$$K(s)\mathbf{y} = \mathbf{u} \tag{7}$$

where $\mathbf{y}$ is the vector of the deviations of the state variables from their fixed point values and $\mathbf{u}$ is the vector of time varying parts of the input signals and K is a 6×6

matrix:

$$K(s) = \begin{bmatrix} k_{11}(s) & 0 & k_{13} & 0 & k_{15} & 0 \\ 0 & k_{22}(s) & 0 & k_{24} & 0 & k_{26} \\ k_{31} & 0 & k_{33}(s) & 0 & 0 & 0 \\ k_{41} & 0 & 0 & k_{33}(s) & 0 & 0 \\ 0 & k_{52} & 0 & 0 & k_{55}(s) & 0 \\ 0 & k_{62} & 0 & 0 & 0 & k_{55}(s) \end{bmatrix} \tag{8}$$

with

$$k_{11}(s) = s + \frac{1}{\tau_e}\left(1 + \frac{\bar{I}_{ee}}{|h_e^{eq} - h_e^{rest}|} + \frac{\bar{I}_{ie}}{|h_i^{eq} - h_e^{rest}|}\right) \tag{9a}$$

$$k_{13} = -\frac{\psi_{ee}(\bar{h}_e)}{\tau_e} \tag{9b}$$

$$k_{15} = -\frac{\psi_{ie}(\bar{h}_e)}{\tau_e} \tag{9c}$$

$$k_{22}(s) = s + \frac{1}{\tau_i}\left(1 + \frac{\bar{I}_{ei}}{|h_e^{eq} - h_i^{rest}|} + \frac{\bar{I}_{ii}}{|h_i^{eq} - h_i^{rest}|}\right) \tag{9d}$$

$$k_{24} = -\frac{\psi_{ei}(\bar{h}_i)}{\tau_i} \tag{9e}$$

$$k_{26} = -\frac{\psi_{ii}(\bar{h}_i)}{\tau_i} \tag{9f}$$

$$k_{31} = -A_{ee}S_e^{'}(\bar{h}_e) \tag{9g}$$

$$k_{33}(s) = (s + \gamma_e)^2 \tag{9h}$$

$$k_{41} = -A_{ei}S_e^{'}(\bar{h}_e) \tag{9i}$$

$$k_{52} = -A_{ie}S_i^{'}(\bar{h}_i) \tag{9j}$$

$$k_{55}(s) = (s + \gamma_i)^2 \tag{9k}$$

$$k_{62} = -A_{ii}S_i^{'}(\bar{h}_i) \tag{9l}$$

The spectrum of interest is proportional to the (1,3) element of the inverse of K evaluated along the imaginary axis of s. This can be written as the quotient:

$$\hat{S}(\omega) = \left|\frac{\det(H(i\omega)}{\det(K(i\omega)}\right|^2 \tag{10}$$

where H is the (3,1) minor of K:

$$H(s) = \begin{bmatrix} 0 & k_{13} & 0 & k_{15} & 0 \\ k_{22}(s) & 0 & k_{24} & 0 & k_{26} \\ 0 & 0 & k_{33}(s) & 0 & 0 \\ k_{52} & 0 & 0 & k_{55} & 0 \\ k_{62} & 0 & 0 & 0 & k_{55}(s) \end{bmatrix} \tag{11}$$

Now, using Matlab for symbolic manipulations, we get:

$$\det K = -k_{33}(s)k_{55}(s)[\{k_{22}(s)k_{55}(s) - k_{26}k_{62}\}\{k_{13}k_{31} - k_{11}(s)k_{33}(s)\} + k_{15}k_{24}k_{41}k_{52}]$$

(12)

and

$$\det H = -k_{13}k_{33}(s)k_{55}(s)\{k_{22}(s)k_{55}(s) - k_{26}k_{62}\} \tag{13}$$

In $\hat{S}(\omega)$, the second order zeros $k_{33}(s)$ and $k_{55}(s)$ in det H cancel the corresponding second order poles in det K, leading to

$$\hat{S}(\omega) = \left| \frac{k_{13}\{k_{22}(i\omega)k_{55}(i\omega) - k_{26}k_{62}\}}{[\{k_{22}(i\omega)k_{55}(i\omega) - k_{26}k_{62}\}\{k_{13}k_{31} - k_{11}(i\omega)k_{33}(i\omega)\} + k_{15}k_{24}k_{41}k_{52}]} \right|^2 \tag{14}$$

Examining the structure of equation (14) it becomes apparent that the power spectral density depends on only 8 distinct (albeit complex) combinations of the low-level original parameters; these are the two rate constants $p_7 = \gamma_e$ and $p_8 = \gamma_i$ and the combinations:

$$p_1 = \frac{1}{\tau_e}(-1 - \frac{\bar{I}_{ee}}{|h_e^{eq} - h_e^{rest}|} - \frac{\bar{I}_{ie}}{|h_i^{eq} - h_e^{rest}|}) \tag{15a}$$

$$p_2 = \frac{1}{\tau_i}(-1 - \frac{\bar{I}_{ei}}{|h_e^{eq} - h_i^{rest}|} - \frac{\bar{I}_{ii}}{|h_i^{eq} - h_i^{rest}|}) \tag{15b}$$

$$p_3 = \frac{\psi_{ee}(\bar{h}_e)}{\tau_e} \tag{15c}$$

$$p_4 = \frac{\psi_{ee}(\bar{h}_e)}{\tau_e} A_{ee} S_e^{'}(\bar{h}_e) = p_3 A_{ee} S_e^{'}(\bar{h}_e) \tag{15d}$$

$$p_5 = \frac{\psi_{ii}(\bar{h}_i)}{\tau_i} A_{ii} S_i^{'}(\bar{h}_i) \tag{15e}$$

$$p_6 = \frac{\psi_{ie}(\bar{h}_e)}{\tau_e} \frac{\psi_{ei}(\bar{h}_i)}{\tau_i} A_{ei} S_e^{'}(\bar{h}_e) A_{ie} S_i^{'}(\bar{h}_i) \tag{15f}$$

Note that many of the terms here (those written with an overbar) are implicitly functions of all the parameters via their dependence on the solutions of the fixed point equations.

In terms of these parameters the PSD becomes:

$$S(\omega) = |\frac{p_3\{(i\omega - p_2)(i\omega + p_8)^2 - p_5\}}{[\{(i\omega - p_2)(i\omega + p_8)^2 - p_5\}\{p_4 - (i\omega - p_1)(i\omega + p_7)^2\} + p_6]}|^2 \tag{16}$$

In addition it is evident that p_3 affects only the total power of the spectrum, and is not directly accessible without detailed information about the source signal and volume conduction effects; in effect it is normalised out in fitting the spectrum to measured data. Thus there remain at most 7 distinct combinations of parameters determining the

form of the PSD. Even in the most favourable case, in which these 7 combinations involve only 7 distinct physiological parameters, there would be at least 15 structurally unidentifiable parameters.

So, consistent with the numerical results, the spectrum is a rational form with 6 poles and 3 zeros rather than 10 poles and 7 zeros as might be expected from simple power counting.

The structure of the transfer function, $T(s)$, is (to within an overall sign) that of a simple feedback system involving two third order filters:

$$T(s) = \frac{H_1(s)}{1 + H_1(s)H_2(s)} \tag{17a}$$

$$H_1(s) = \frac{k_{13}}{k_{13}k_{31} - k_{11}(s)k_{33}(s)} \tag{17b}$$

$$H_2(s) = \frac{k_{15}k_{24}k_{41}k_{52}}{k_{13}\{k_{22}(s)k_{55}(s) - k_{26}k_{62}\}} \tag{17c}$$

$$\hat{S}(\omega) = |T(i\omega)|^2 \tag{17d}$$

In this case, the pole locations of H_1 are governed mainly by properties of excitatory cells while those of H_2 by the properties of inhibitory cells. In practice, H_1 appears to be a low-pass filter and H_2 is resonant.
