# Supplementary material for: Parameter estimation and identifiability in a neural population model for electro-cortical activity
Source: PLoS Comput Biol. 2019 May 30;15(5):e1006694. doi: 10.1371/journal.pcbi.1006694 (PMC6542506; doi:10.1371/journal.pcbi.1006694)
Supplement: S2 Appendix — The derivation of Eq (24) from Eq (23). (PDF) [file pcbi.1006694.s002.pdf]

S2 Appendix

Derivation of the Fisher Information Matrix

The definition of the Fisher information matrix (FIM) for a p.d.f. $f(\mathbf{x}|\boldsymbol{\theta})$ is :

$$I_{\mu\nu}(\boldsymbol{\theta}) = \int f(\mathbf{x}|\boldsymbol{\theta}) \frac{\partial \ln f(\mathbf{x}|\boldsymbol{\theta})}{\partial \theta_\mu} \frac{\partial \ln f(\mathbf{x}|\boldsymbol{\theta})}{\partial \theta_\nu} d^N\mathbf{x} \tag{1}$$

If the components of the vector $\mathbf{x}$ are independent then

$$f(\mathbf{x}|\boldsymbol{\theta}) = \prod_k f_k(x_k|\boldsymbol{\theta}) \tag{2}$$

For this case, taking account of the normalisation, $\int f_k(x_k|\boldsymbol{\theta})dx_k = 1$, it is straightforward to show that:

$$I_{\mu\nu}(\boldsymbol{\theta}) = \sum_k I^{(k)}_{\mu\nu}(\boldsymbol{\theta}) \tag{3}$$

where

$$I^{(k)}_{\mu\nu}(\boldsymbol{\theta}) = \int f_k(x_k|\boldsymbol{\theta}) \frac{\partial \ln f_k(x_k|\boldsymbol{\theta})}{\partial \theta_\mu} \frac{\partial \ln f_k(x_k|\boldsymbol{\theta})}{\partial \theta_\nu} dx_k \tag{4}$$

is the FIM based on the marginal p.d.f. for the component x_k.

For the problem in which the components of the vector $\mathbf{x}$ are model spectral estimates at different frequencies evaluated using the Welch periodogram, and providing that the correlations introduced by the overlap and non-uniform shapes of the windows used can be neglected, the spectral estimate at a given frequency has a gamma distribution of the form:

$$f_k(x_k; K, \Theta_k) = \frac{x_k^{K-1} e^{-\frac{x_k}{\Theta_k}}}{\Theta_k^K \Gamma(K)}; x_k \geq 0 \tag{5}$$

where K is the number of segments averaged in the Welch periodogram (which is independent of the system parameters, $\boldsymbol{\theta}$) and the scale parameter, Θ_k, (which does depend on the system parameters) is related to the model's expected power spectral density at the given frequency by

$$\Theta_k = \frac{\alpha |T(i\omega_k|\boldsymbol{\theta})|^2}{K} \tag{6}$$

In this equation, $T(s|\boldsymbol{\theta})$ is the linearized model transfer function, ω_k is the kth sampled frequency and α is the power matching parameter introduced in the main article. For our immediate purpose, investigating the sensitivity of the model output to changes in the system parameters, we can treat α as a given constant. With these distributions the integrals in the definition of the Fisher information matrix can be explicitly performed and the expression for $I^{(k)}_{\mu\nu}(\boldsymbol{\theta})$ considerably simplified. Thus (dropping the k subscripts for convenience):

$$\ln f(x; K, \Theta) = (K-1)\ln x - \frac{x}{\Theta} - K \ln \Theta - \ln \Gamma(K) \tag{7}$$

so

$$\frac{\partial}{\partial \theta_\mu} \ln f(x; K, \Theta) = (x - K\Theta) \frac{1}{\Theta^2} \frac{\partial \Theta}{\partial \theta_\mu} \tag{8}$$

Then, substituting for the derivatives and moving terms independent of x outside the integral, we get

$$I_{\mu\nu}(\boldsymbol{\theta}) = \frac{1}{\Theta^4}\frac{\partial\Theta}{\partial\theta_\mu}\frac{\partial\Theta}{\partial\theta_\nu}\int f(x;K,\Theta)(x-K\Theta)^2 dx \tag{9}$$

The mean of the gamma distribution is $K\Theta$ so the integral in the equation is equal to the variance of the gamma distribution, which is equal to $K\Theta^2$, thus:

$$\begin{aligned}
I_{\mu\nu}(\boldsymbol{\theta}) &= \frac{1}{\Theta^4}\frac{\partial\Theta}{\partial\theta_\mu}\frac{\partial\Theta}{\partial\theta_\nu}\ \mathrm{var}(X) \\
&= \frac{K}{\Theta^2}\frac{\partial\Theta}{\partial\theta_\mu}\frac{\partial\Theta}{\partial\theta_\nu} \\
&= K\frac{\partial\ln\Theta}{\partial\theta_\mu}\frac{\partial\ln\Theta}{\partial\theta_\nu} \\
&= K\frac{\partial\ln|T(i\omega|\boldsymbol{\theta})|^2}{\partial\theta_\mu}\frac{\partial\ln|T(i\omega|\boldsymbol{\theta})|^2}{\partial\theta_\nu}
\end{aligned} \tag{10}$$

Thus, restoring the k subscripts and defining the sampled values of the model power spectral density, $\hat{S}_k \equiv |T(i\omega_k|\boldsymbol{\theta})|^2$, we get

$$I_{\mu\nu}(\boldsymbol{\theta}) = K\sum_k \frac{\partial\ln\hat{S}_k}{\partial\theta_\mu}\frac{\partial\ln\hat{S}_k}{\partial\theta_\nu} \tag{11}$$

Since, in the region of interest, the transfer function for the linearized model appears to have no zeros on the imaginary axis, it follows that $\hat{S}_k > 0$ and this form provides a useful simplification of the FIM.
