# Supplementary material for: Parameter estimation and identifiability in a neural population model for electro-cortical activity
Source: PLoS Comput Biol. 2019 May 30;15(5):e1006694. doi: 10.1371/journal.pcbi.1006694 (PMC6542506; doi:10.1371/journal.pcbi.1006694)
Supplement: S3 Appendix — Computation and analysis of the directional derivatives of the modelled spectra along the directions of the leading eigenvectors. (PDF) [file pcbi.1006694.s003.pdf]

S3 Appendix

Directional derivatives

In order to provide a basis for speculation about the roles played by the various parameter combinations identified by the eigenvectors of the FIM, we have calculated the directional derivatives of the modelled spectra along the directions of the leading eigenvectors. The derivatives are defined by

$$\frac{dS(f)}{d\boldsymbol{e_n}} = \sum_{m=1}^{N} e_{nm} \frac{\partial S(f)}{\partial \theta_m}; n = 1, 2, \cdots, 22 \tag{1}$$

where $\boldsymbol{e_n}$ is the normalised eigenvector of the FIM in order of decreasing eigenvalue (i.e. $\lambda_{n+1} \leq \lambda_n$). (Note that the FIM eigenvectors are calculated on the basis of normalized parameters, and are adjusted to yield the corresponding vectors in the raw parameter space before being used in this equation.)

Only the first 3 vectors yield reasonably straightforward interpretations as shown in Fig 1, so we have limited our analysis to these directions. A basis for interpreting these results can be found by considering the directional derivatives of a localised single peaked distribution with respect to its location, relative height (w.r.t. an independent background) and its scale. Fig 2 shows the corresponding derivatives for a simple Gaussian shape,

$$S(f) = Ae^{-\frac{1}{2}a(f-f_0)^2} \tag{2}$$

but other similarly peaked functions can be expected to behave similarly.

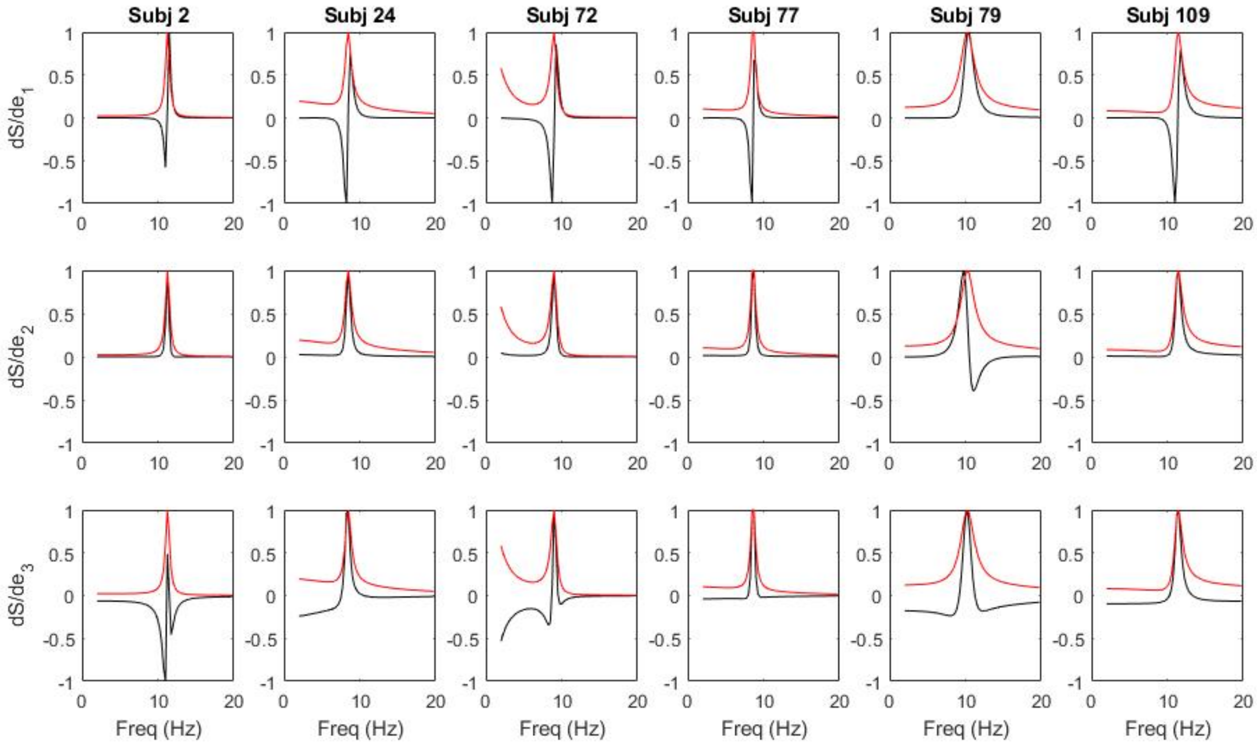

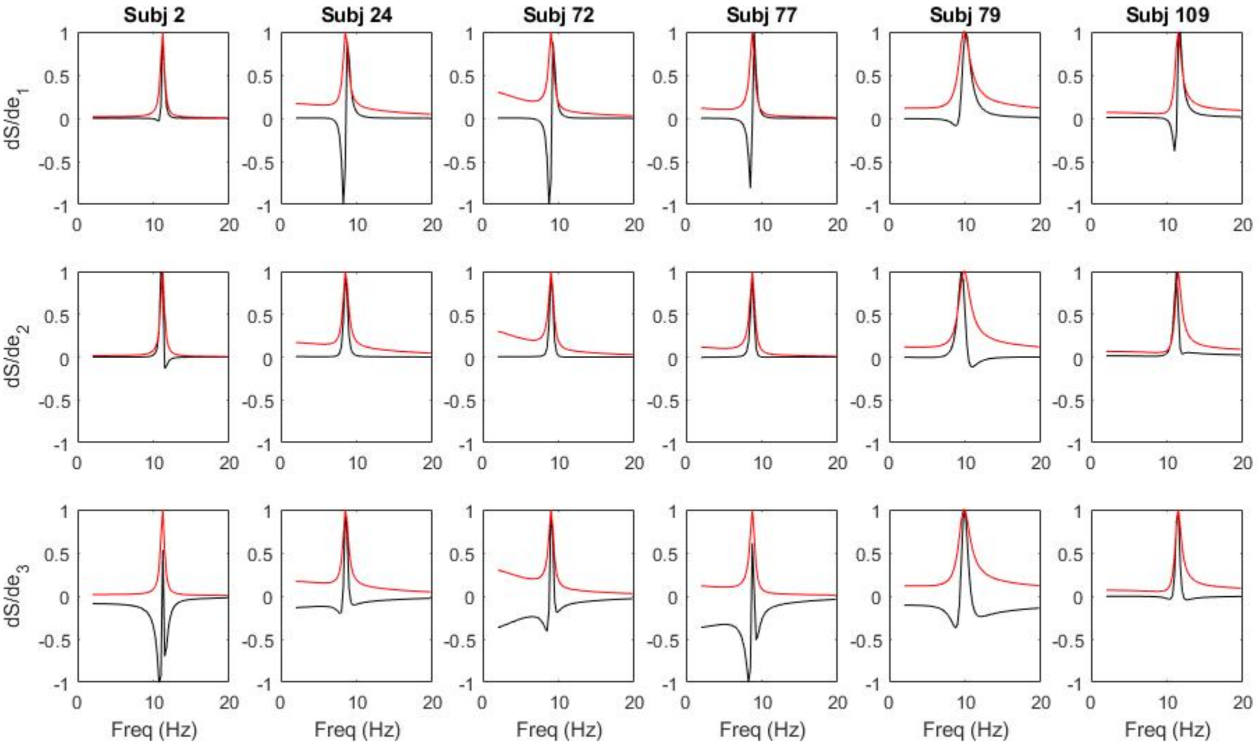

Fig 1. Directional derivatives. The directional derivatives of the modelled spectra (black) with respect to the three leading FIM eigenvector directions are plotted as functions of frequency. The red curves are the corresponding modelled spectra. Both the spectra and the derivatives are evaluated at the LS parameters (top 3 rows) and the ML parameters (bottom 3 rows) determined for the 6 chosen example subjects used in the main article. For convenient comparison all curves have been normalised to fit within the range -1 to +1.

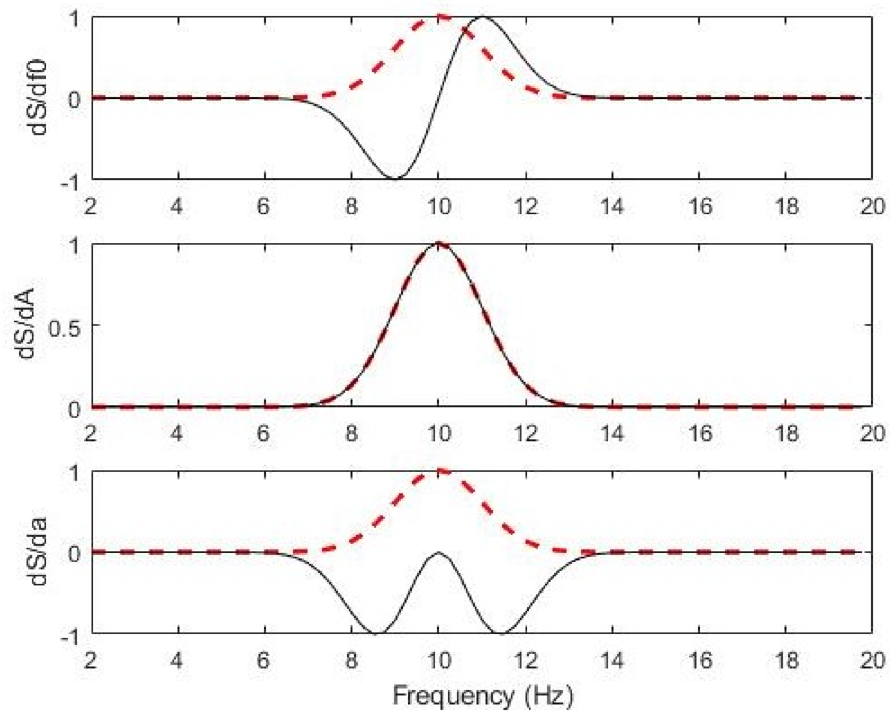

Fig 2. Derivatives of a Gaussian shape w.r.t. some features. Derivatives of a Gaussian shape w.r.t. peak position (top), amplitude (middle) and scale (bottom). (The Gaussian is shown as a dashed red line and the derivative as a thin black line.)
