# Supplementary material for: Parameter estimation and identifiability in a neural population model for electro-cortical activity
Source: PLoS Comput Biol. 2019 May 30;15(5):e1006694. doi: 10.1371/journal.pcbi.1006694 (PMC6542506; doi:10.1371/journal.pcbi.1006694)
Supplement: S1 Fig — Comparisons of time series of the EEG data and representative samples of modelled time series using parameters for the 6 subjects shown in Figs 1 and 2. (PDF) [file pcbi.1006694.s004.pdf]

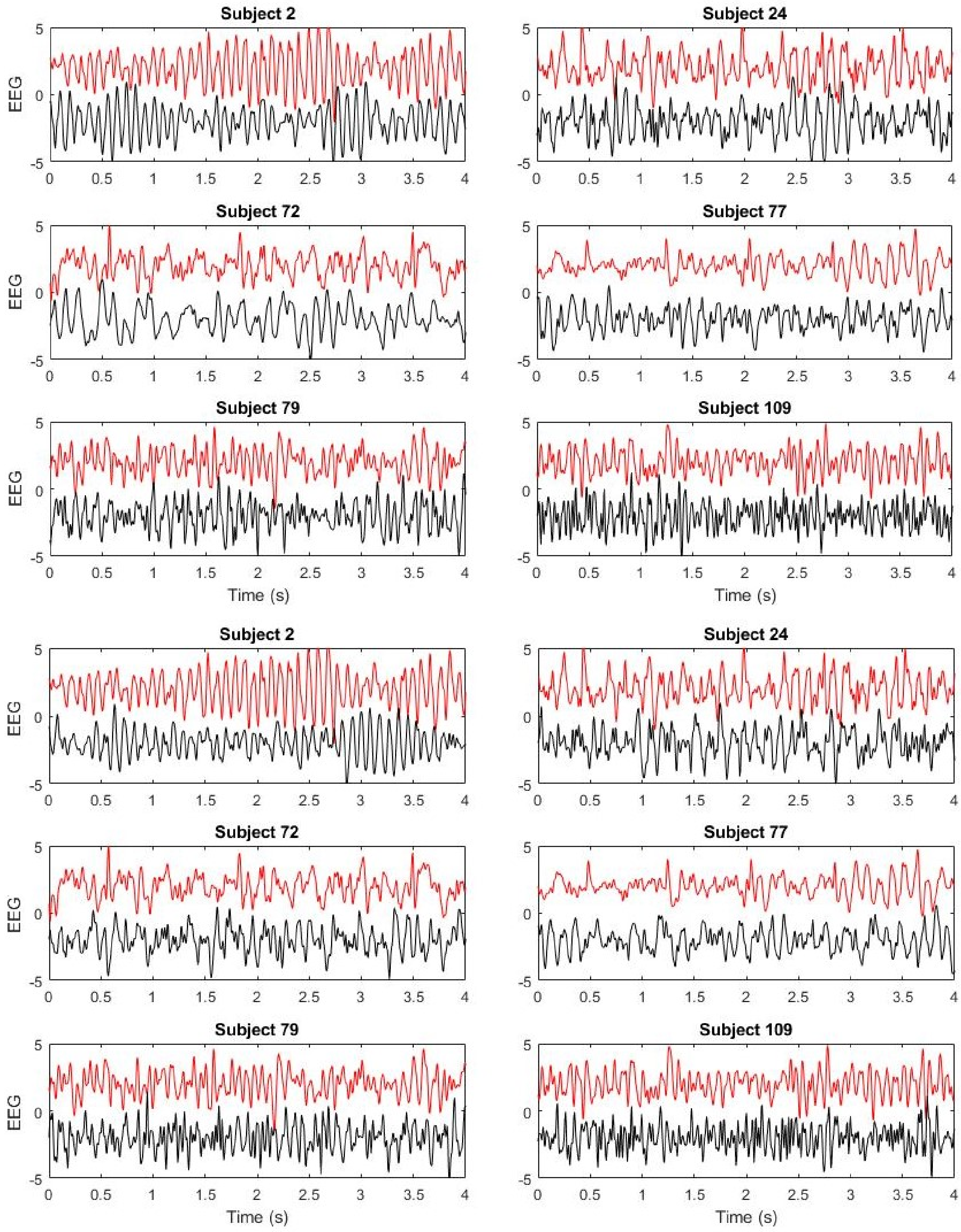


**S1 Fig. Time series of data and modelled system output.** Comparisons of time series of the EEG data (red curves) and representative samples of modelled time series using parameters (black curves) for the 6 subjects shown in Figures 1 and 2. The top 3 rows use the LS parameters and the bottom 3 use the ML parameters. The EEG data and modelled output have been normalised to their respective mean square values and are shown offset for convenient comparison. Across the full population (results not shown here) the waveforms of the corresponding time series are usually similar, although some deviations are apparent in the case of less typical EEG (e.g. Subject 77). Note that, in order to avoid excess un-modelled frequency components, both the raw EEG and the modelled output have been filtered between 2 and 40 Hz using a linear-phase, equi-ripple FIR filter with 60dB attenuation in the stop bands and transition bands of 2Hz.
