# Supplementary material for: Parameter estimation and identifiability in a neural population model for electro-cortical activity
Source: PLoS Comput Biol. 2019 May 30;15(5):e1006694. doi: 10.1371/journal.pcbi.1006694 (PMC6542506; doi:10.1371/journal.pcbi.1006694)
Supplement: S2 Fig — The eigenspectra of the Fisher information matrices plotted for all 82 subjects. (PDF) [file pcbi.1006694.s005.pdf]

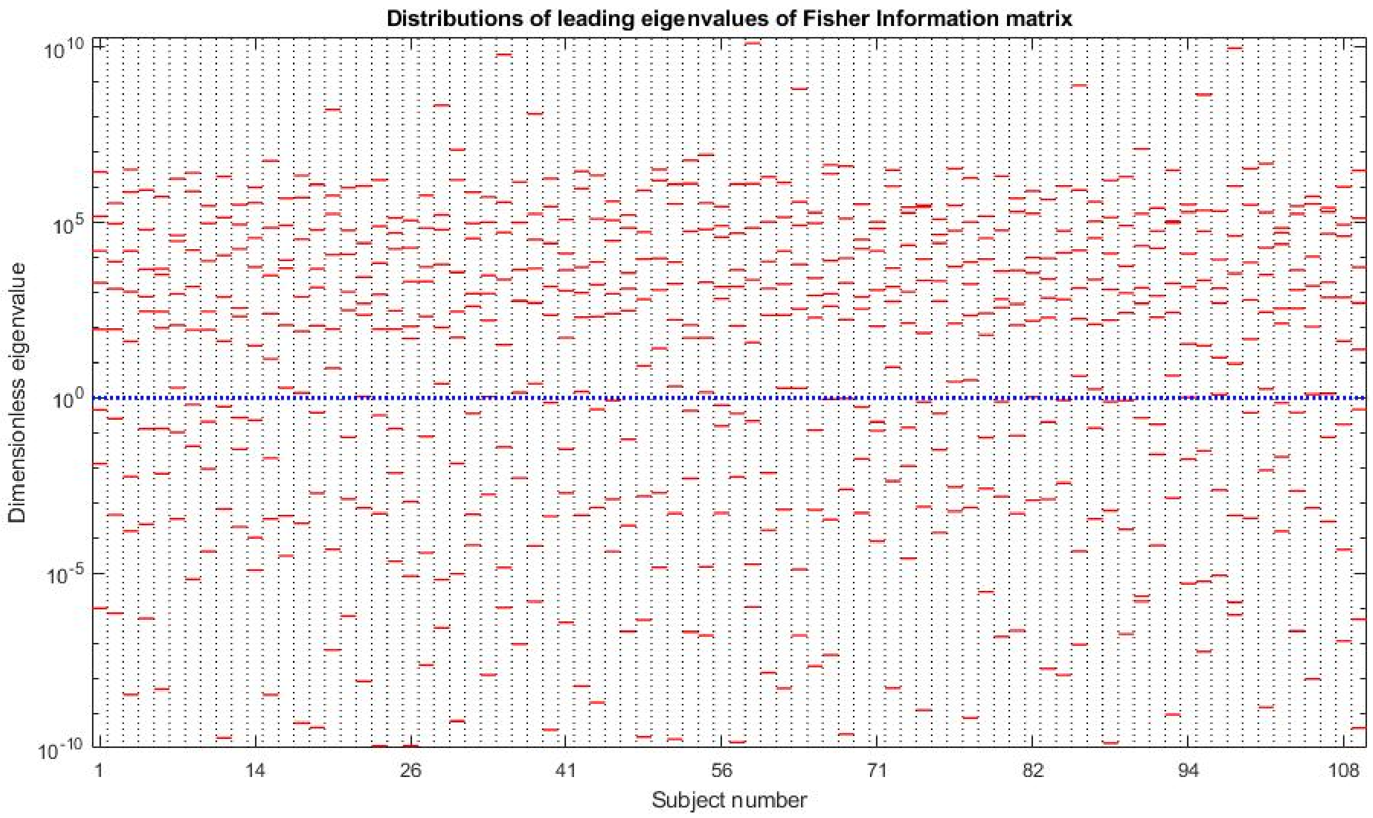

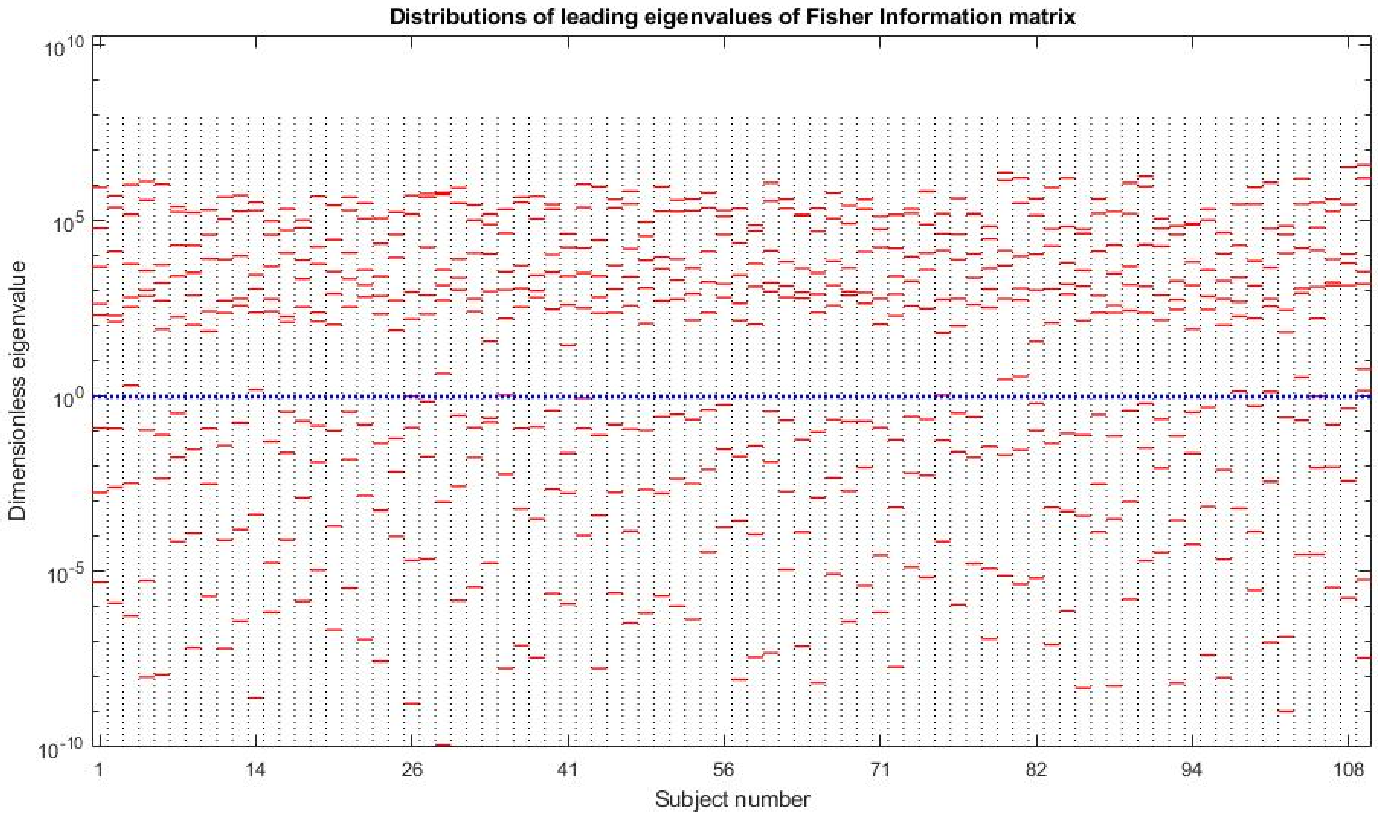

S2 Fig. FIM eigenspectra for all subjects. The spectra of the Fisher information matrices have been plotted here for all 82 subjects; the top plot using LS parameters and the bottom plot using the ML parameters. The similarity of the character of the spectra across most subjects is apparent.
