# Supplementary material for: Parameter estimation and identifiability in a neural population model for electro-cortical activity
Source: PLoS Comput Biol. 2019 May 30;15(5):e1006694. doi: 10.1371/journal.pcbi.1006694 (PMC6542506; doi:10.1371/journal.pcbi.1006694)
Supplement: S3 Fig — The distribution showing how different values of each decile of the relative costs are spread across the 82 subjects. (PDF) [file pcbi.1006694.s006.pdf]

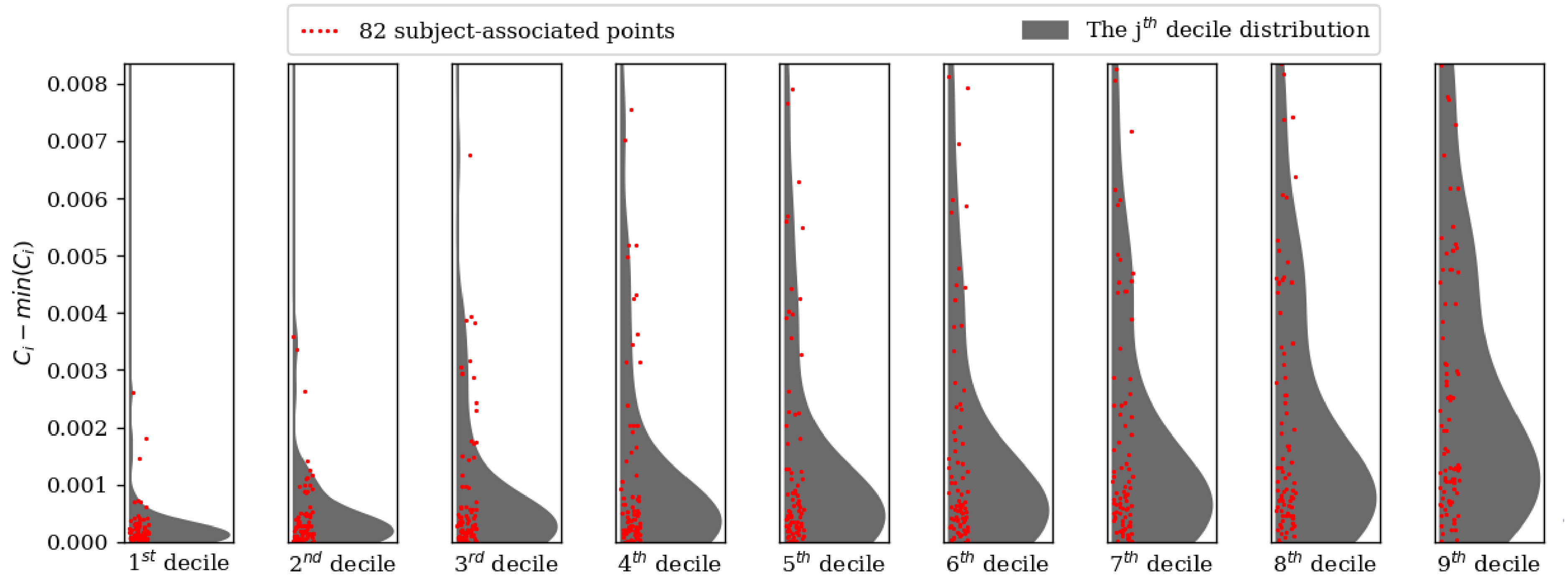

S3 Fig. Distribution of cost functions found by particle swarm optimization (PSO). In the PSO approach, the spectrum from each subject, i (where i $= 1 \ldots 82$), is independently fit 1000 times, giving 1000 different parameter estimates (where each estimate is a 22-dimensional vector). However, several of these estimates are far from the global optimum and give visually poor fits. To decide on an objective criterion for which fits to keep, we examined the distribution of cost functions C_{ij} for each of the 1000 different estimates (j) in each of the 82 subjects (i). The 'relative cost', $C_{ij} - min(C_i)$ is the difference between the cost from a given sample and the minimum cost for that subject and is a measure of relative fit quality. On the leftmost plot, the relative cost of the estimate with the 100th lowest cost function (the 1st decile) is shown for each of the 82 subjects (red dots). The next plot shows the relative cost of the estimate with the 200th smallest cost function (the 2nd decile), and so on. These decile plots highlight the presence of outliers ('bad fits') which become more extreme and more frequent as a greater proportion of the estimates are included away from the global optimum. By selecting only those fits in the first decile —the top 10% of fits to each subject's spectrum, in other words, the 100 best fits— we minimize the influence of outliers while still preserving a sufficient number of samples to characterize the shape of the cost function near the global optimum.
