# Supplementary material for: Parameter estimation and identifiability in a neural population model for electro-cortical activity
Source: PLoS Comput Biol. 2019 May 30;15(5):e1006694. doi: 10.1371/journal.pcbi.1006694 (PMC6542506; doi:10.1371/journal.pcbi.1006694)
Supplement: S4 Fig — Absolute values of correlation coefficients and selected pairwise distributions based on MCMC parameter distributions for the 6 selected subjects. (PDF) [file pcbi.1006694.s007.pdf]

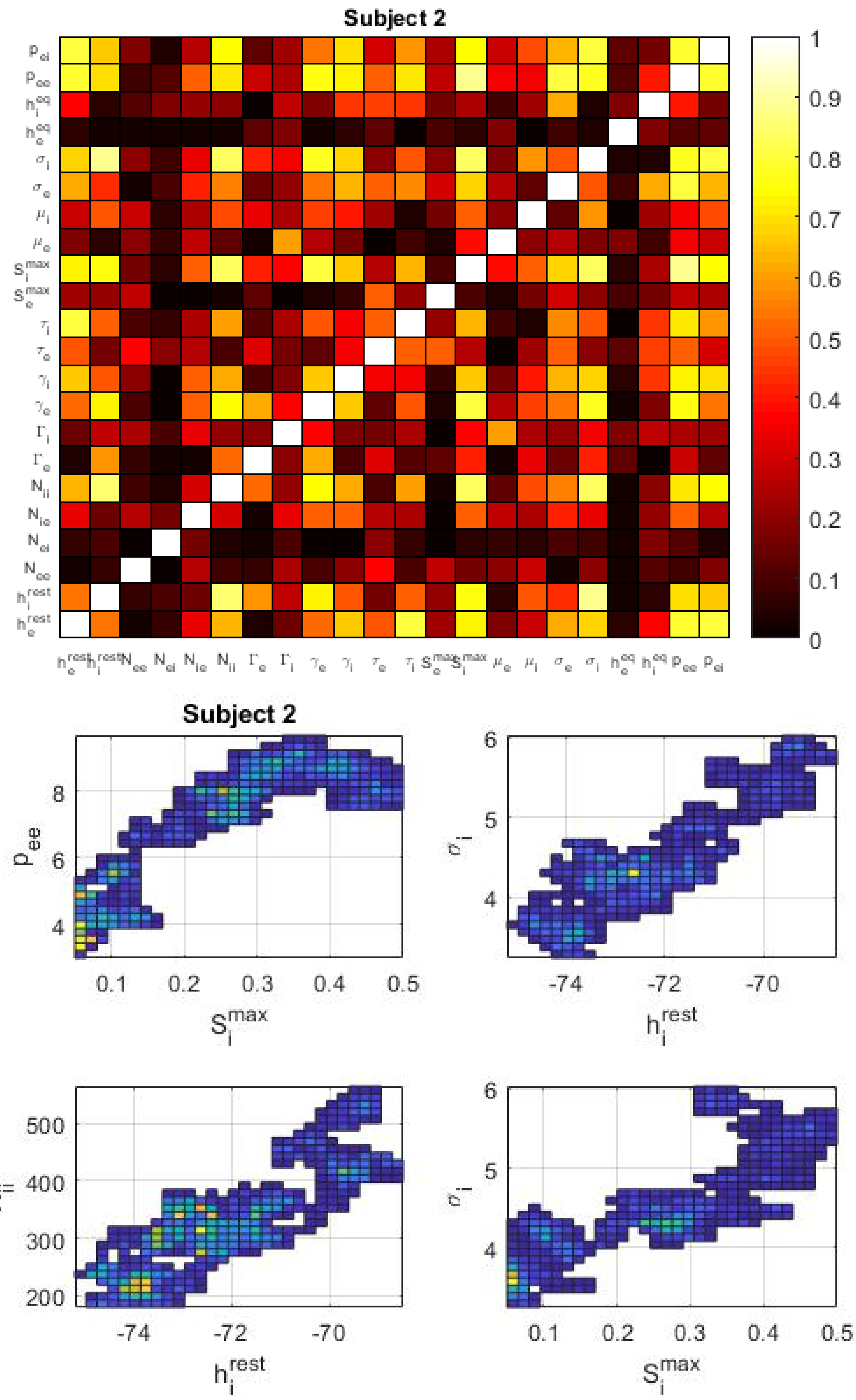

S4^a Fig. Correlation matrices and pairwise distributions^a. Absolute values of correlation coefficients and selected pairwise distributions based on MCMC parameter distributions for Subject 2. Because of the large number of pairs involved and the complexity of their interrelation as well as the variability across subjects shown in S4^a Fig - S4^f Fig, these are of little use in suggesting meaningful combinations of parameters. Indeed, stronger correlations are associated with “sloppy” rather than”stiff” directions in parameter space.

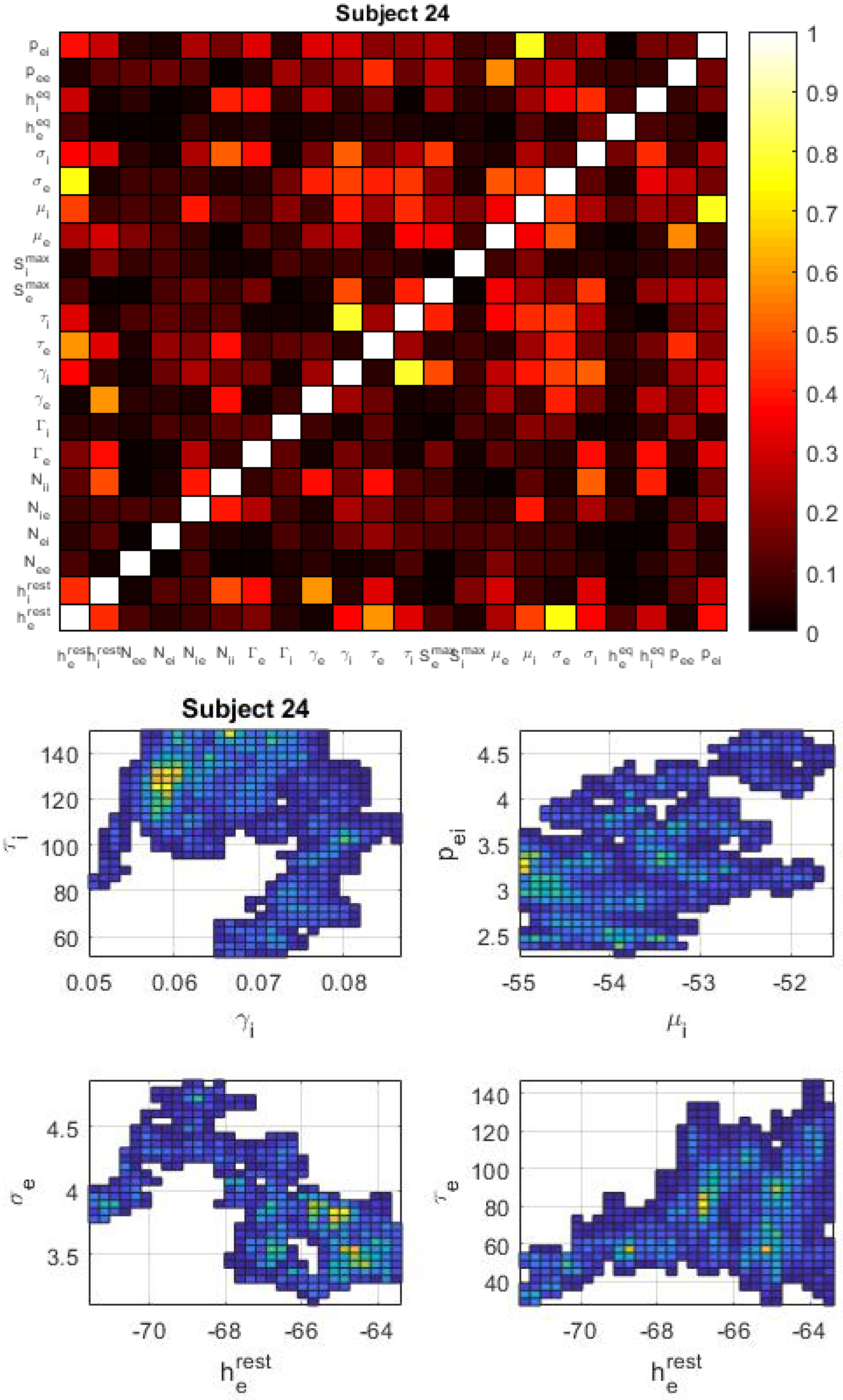

S4^b Fig. Correlation matrices and pairwise distributions^b. Absolute values of correlation coefficients and selected pairwise distributions based on MCMC parameter distributions for Subject 24.

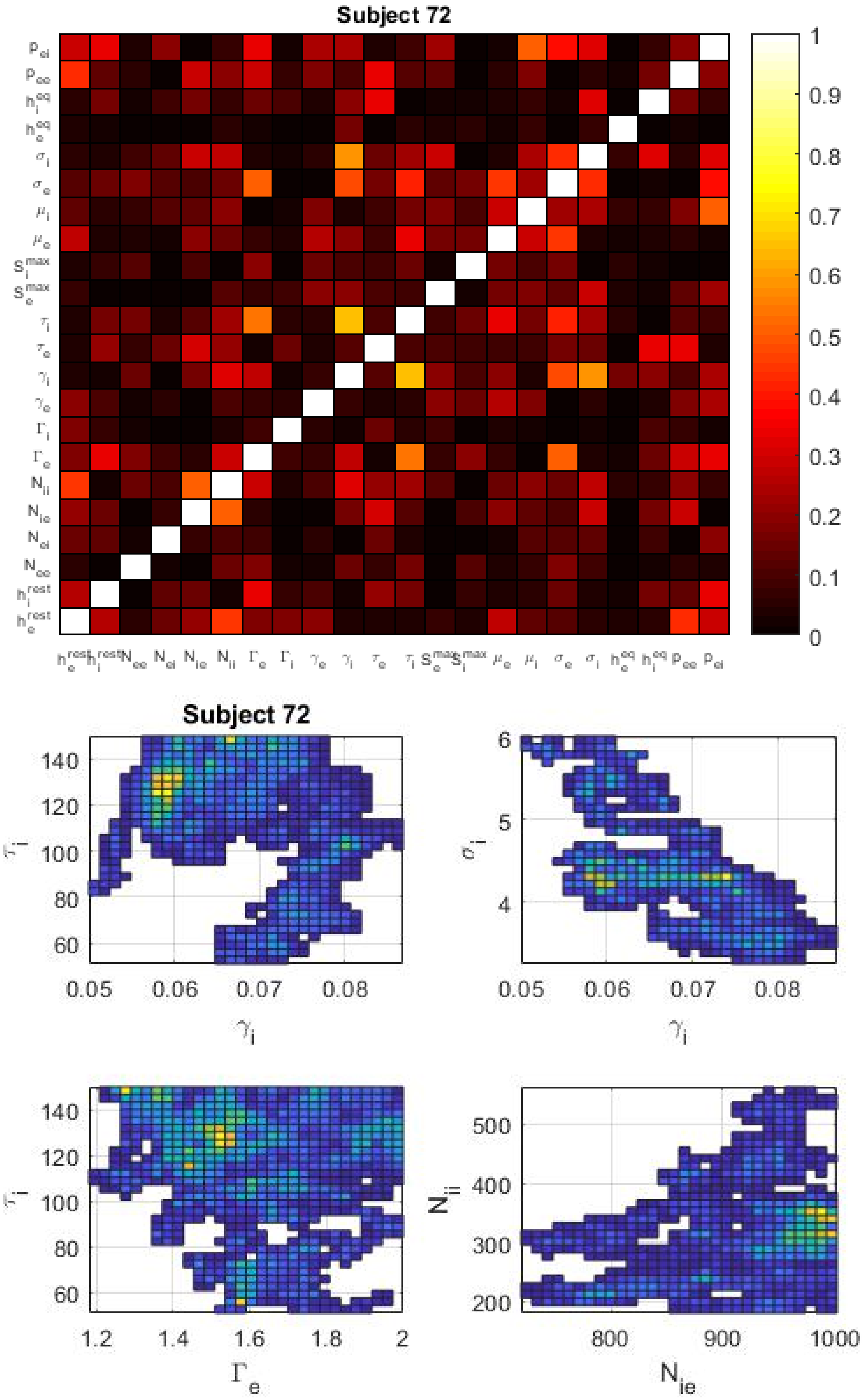

S4[c] Fig. Correlation matrices and pairwise distributions[c]. Absolute values of correlation coefficients and selected pairwise distributions based on MCMC parameter distributions for Subject 72.

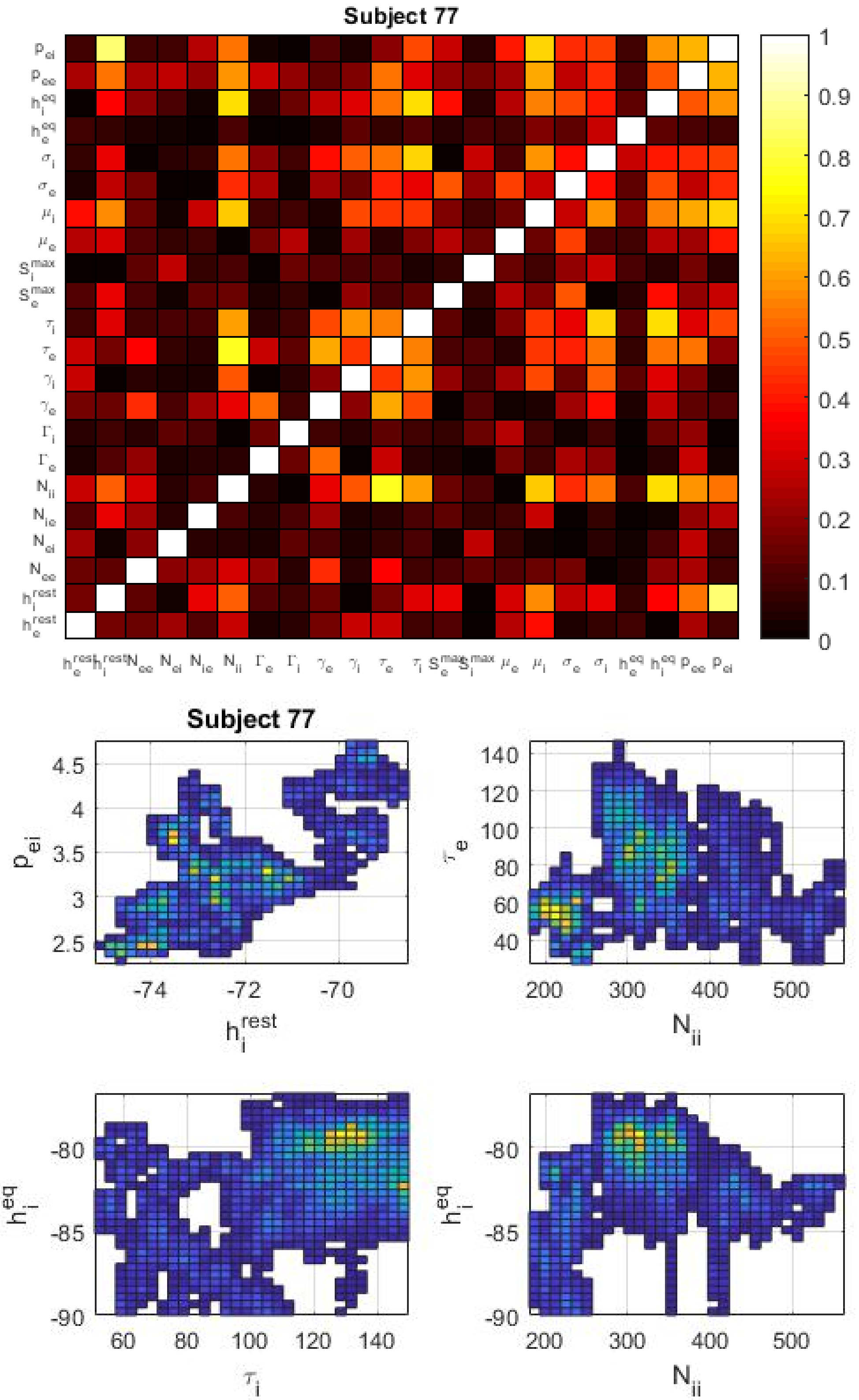

S4[d] Fig. Correlation matrices and pairwise distributions[d]. Absolute values of correlation coefficients and selected pairwise distributions based on MCMC parameter distributions for Subject 77.

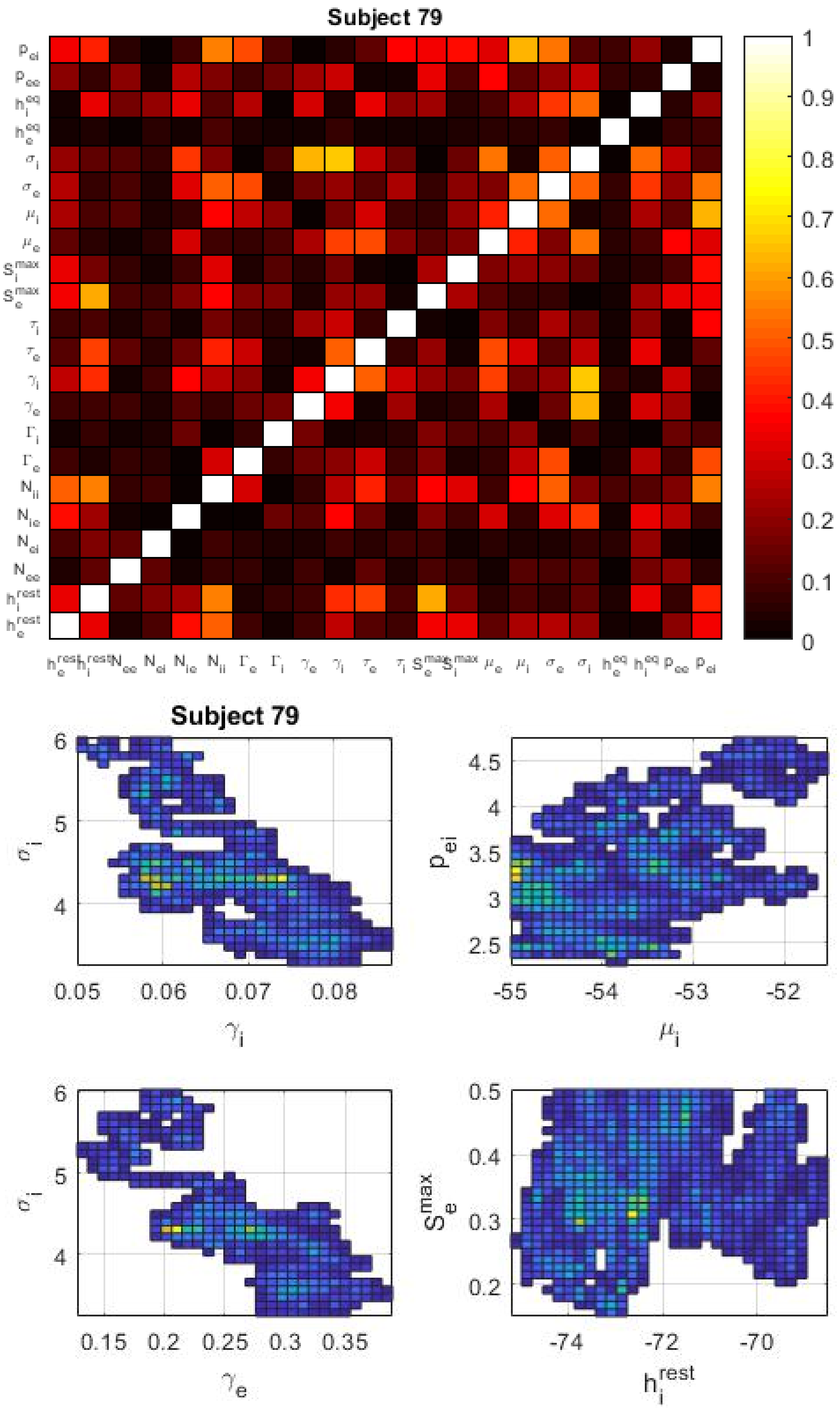

S4[e] Fig. Correlation matrices and pairwise distributions[e]. Absolute values of correlation coefficients and selected pairwise distributions based on MCMC parameter distributions for Subject 79.

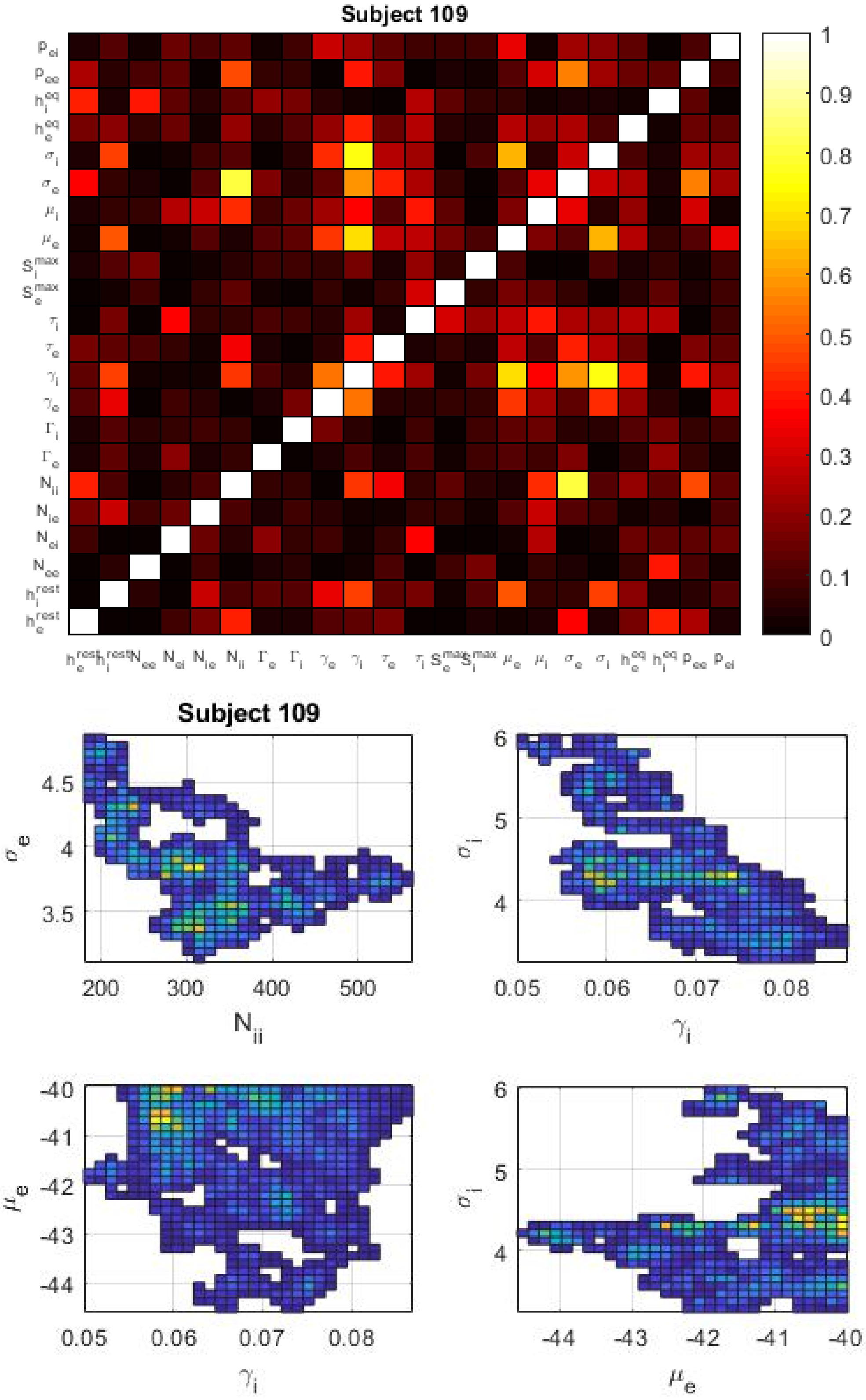

S4[f] Fig. Correlation matrices and pairwise distributions[f]. Absolute values of correlation coefficients and selected pairwise distributions based on MCMC parameter distributions for Subject 109.
