# Supplementary material for: Parameter estimation and identifiability in a neural population model for electro-cortical activity
Source: PLoS Comput Biol. 2019 May 30;15(5):e1006694. doi: 10.1371/journal.pcbi.1006694 (PMC6542506; doi:10.1371/journal.pcbi.1006694)
Supplement: S5 Fig — Pseudocolor plots of the ratios of the magnitudes of the components to the L1 norm of the vector for the leading eigenvectors of the FIM (ML parameters) for the full set of 82 subjects. (PDF) [file pcbi.1006694.s008.pdf]

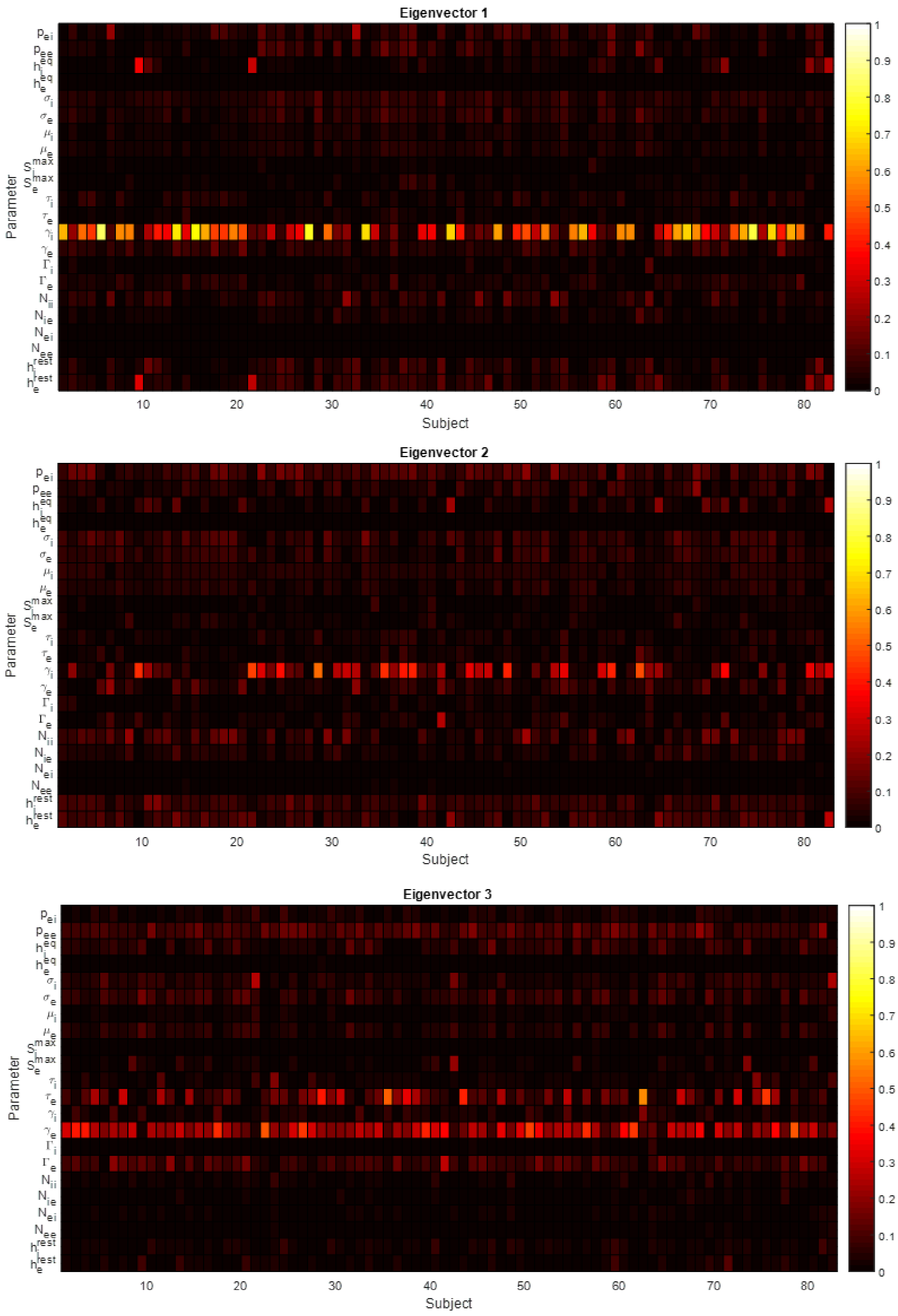

S5[a] Fig. Relative magnitudes of the components of the leading eigenvectors across subjects[a]. Pseudocolor plots of the ratios of the magnitudes of the components to the L1 norm of the vector for the 3 leading eigenvectors of the FIM (ML parameters) for the full set of 82 subjects. The top panel is for the 1^{st}(largest eigenvalue) eigenvector, the middle for the 2nd and the bottom for the 3rd. The gross features of the relative magnitudes in any vector appear to be relatively robust across subjects, although not universally so. The pattern of increasing complexity of the combination with decreasing eigenvalue is also apparent. A tentative interpretation of the roles played by these 3 combinations is given in S3 Appendix.

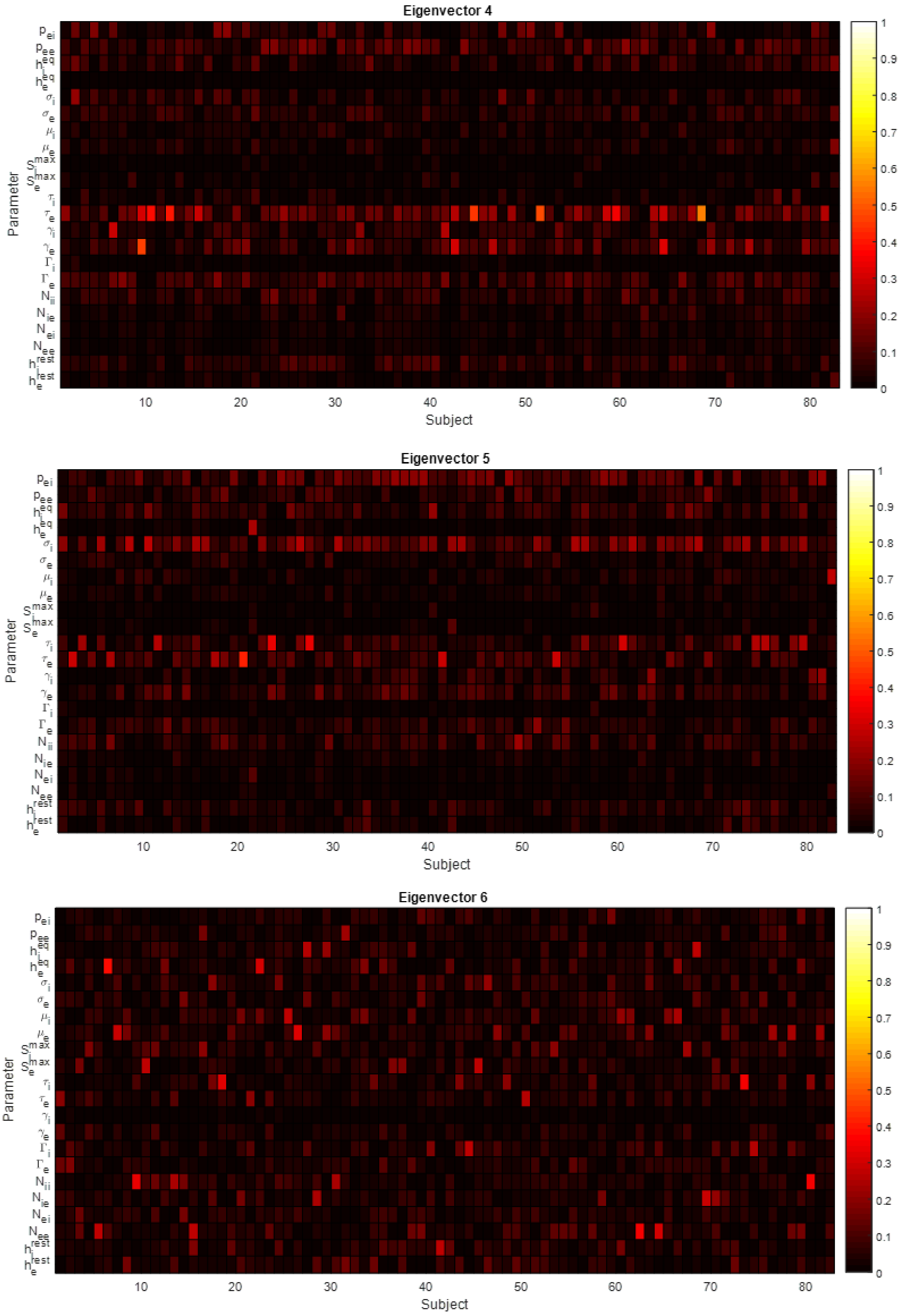

S5[b] **Fig. Relative magnitudes of the components of the leading eigenvectors across subjects**[b]**.** As for S5[a] Figure, but for the 4th, 5th and 6th eigenvectors. The pattern of increasing complexity of the combinations with decreasing eigenvalue is still apparent, but the patterns are less robust across subjects, tending to independence for eigenvectors beyond the 5th.
